# Prevalence, Antibiotic Resistance, and Biofilm Formation of *Proteus mirabilis* in Dairy Products: Implications for Veterinary and Public Health

**DOI:** 10.1002/vms3.70600

**Published:** 2025-09-29

**Authors:** Afrooz Shafiei Lordejani, Elaheh Tajbakhsh, Faham Khamesipour, Hassan Momtaz, Manocher Momeni Shahraki

**Affiliations:** ^1^ Department of Biology ShK.C., Islamic Azad University Shahrekord Iran; ^2^ Halal Research Center of the Islamic Republic of Iran (IRI), Iran Food and Drug Administration Ministry of Health and Medical Education Tehran Iran; ^3^ Department of Veterinary ShK.C., Islamic Azad University Shahrekord Iran

**Keywords:** biofilms, dairy products, drug resistance, food contamination, microbial, *Proteus mirabilis*, veterinary public health

## Abstract

Dairy products are essential components of human and animal nutrition, providing vital nutrients such as proteins, vitamins and minerals. However, their susceptibility to microbial contamination, particularly by pathogens like *Proteus mirabilis*, poses significant risks to both animal and public health. This study investigated the prevalence, antibiotic resistance patterns and biofilm‐forming ability of *P. mirabilis* in various dairy products, with a focus on raw milk, in Shahrekord, Iran. A total of 480 samples, including raw cow, goat and sheep milk, as well as cheese, yogurt, cream, curd and a traditional Iranian fermented yogurt‐based beverage (doogh), were analysed under controlled laboratory conditions. The findings revealed a 12.29% prevalence rate of *P. mirabilis*, with raw cow's milk showing the highest contamination rate (21.66%). Among the isolates, 88.12% were capable of forming biofilms, and 71.15% exhibited strong biofilm production. Antibiotic susceptibility testing identified a high prevalence of multidrug‐resistant strains, with the highest resistance rates observed for cotrimoxazole (59.32%) and gentamicin (50.84%). In addition, 25.42% of the isolates were identified as extended‐spectrum beta‐lactamase (ESBL) producers, with *blaCTX‐M* being the most prevalent resistance gene.

A significant correlation was found between biofilm‐forming ability and the presence of antibiotic resistance genes, highlighting the dual challenges of microbial persistence and antimicrobial resistance. These findings emphasize the need for improved hygiene practices in dairy production, targeted biofilm‐disrupting strategies and enhanced surveillance programs to mitigate risks to both animal and public health. This study provides critical insights for veterinary professionals and policymakers to develop effective interventions aimed at reducing contamination and combating antimicrobial resistance in dairy products.

## Graphical abstract

This graphical abstract illustrates the study's core findings on the microbiological safety of dairy products with regard to *Proteus mirabilis*. A total of 480 dairy samples including raw cow, goat and sheep milk, cheese, yogurt, cream, curd and doogh were microbiologically analysed under controlled laboratory conditions in Shahrekord, Iran.

The **prevalence** of *P. mirabilis* was identified in **12.29%** of the samples, with **raw cow's milk** exhibiting the highest contamination rate. Antibiotic susceptibility testing revealed a **high frequency of multidrug‐resistant strains**, notably:

**• 59.32% resistance** to cotrimoxazole,

**• 50.84% resistance** to gentamicin, and

**• 25.42%** of isolates confirmed as ESBL (extended‐spectrum beta‐lactamase) producers.

Furthermore, **88.12% of the isolates** demonstrated the ability to form biofilms, which are structurally complex communities contributing to microbial persistence and resistance. A statistically significant correlation was found between biofilm formation and the presence of antibiotic resistance genes, particularly *blaCTX‐M*.

These findings underscore the urgent need for **enhanced hygiene protocols**, **targeted biofilm‐disrupting interventions** and **comprehensive antimicrobial surveillance** throughout the dairy production and supply chain. The study offers critical insights for public health authorities and veterinary professionals to mitigate microbial risks in animal‐derived food products.

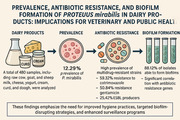

## 1 Introduction

Milk and dairy products are fundamental to both human and animal nutrition, providing essential nutrients such as proteins, vitamins and minerals. However, their susceptibility to microbial contamination poses significant risks to food safety and public health. Among the microbial contaminants, members of the Enterobacteriaceae family, particularly *Proteus mirabilis*, have emerged as pathogens of concern due to their role in foodborne illnesses and their association with spoilage and faecal contamination in food systems (Goyal et al. 2009; Mladenović et al. 2021; Reta et al. 2016; Tian et al. 2024a). The economic and public health implications of such contamination highlight the critical need for monitoring and managing microbial hazards in dairy production, particularly in regions where veterinary and food safety infrastructure may be underdeveloped.

*P. mirabilis*, a Gram‐negative, facultatively anaerobic bacterium, is widely distributed in water, soil and the gastrointestinal tracts of humans and animals. While typically a commensal organism, *P. mirabilis* can transition into an opportunistic pathogen, causing urinary tract infections, wound infections and neonatal meningitis (Ahn et al. 2023; Gao and Tian 2024). Its presence in dairy products, particularly raw milk, has become a growing concern, with cross‐contamination during milking and processing identified as a primary route of entry into the food supply chain (Tian et al. 2024b). This poses substantial risks to both animal and human health, especially in regions where hygiene practices during dairy production may be suboptimal.

A critical feature of *P. mirabilis* is its ability to form biofilms complex microbial communities encased in an extracellular matrix. Biofilm formation enhances bacterial resistance to antibiotics, environmental stressors and sanitization efforts, making *P. mirabilis* a formidable challenge in dairy processing environments (Meng et al. 2021; Palmer et al. 2021; Wasfi et al. 2021). The regulatory mechanisms driving biofilm development involve various virulence‐associated genes, such as *ureC*, *zapA* and *rsmA*, which govern adhesion, motility and extracellular polymeric substance production (Gao and Tian 2024). These biofilms not only protect *P. mirabilis* but also facilitate its persistence and transmission in dairy systems, amplifying its potential as a zoonotic and public health threat.

The threat posed by *P. mirabilis* is further exacerbated by its increasing antibiotic resistance, particularly the emergence of multidrug‐resistant (MDR) strains. The overuse of antibiotics in veterinary and agricultural practices has accelerated the development of these resistant strains, which have been isolated from both clinical and food sources (Chong et al. 2013; Song et al. 2011; Wu et al. 2024a; Wu et al. 2024b). MDR *P. mirabilis* represents a dual burden: complicating treatment in veterinary and healthcare settings and increasing the risk of transmission through contaminated food products. Recent advances in molecular biology, including transcriptomic and metabolomic analyses, have identified potential therapeutic targets to combat biofilm formation and resistance, such as pathways involved in flagellar assembly and lipopolysaccharide synthesis (Gao and Tian 2024; Tian et al. 2024b).

Despite its significance, comprehensive data on the prevalence, biofilm‐forming capabilities, and resistance profiles of *P. mirabilis* in dairy products remain scarce, particularly in developing regions where veterinary and food safety infrastructure may be inadequate. This study seeks to address these gaps by investigating the distribution of biofilm‐associated, resistance and virulence genes in *P. mirabilis* isolates from milk and dairy products in Iran. By elucidating the molecular mechanisms underlying its pathogenicity and resilience, this research aims to contribute to the development of targeted strategies for mitigating its impact on animal health, food safety and public health.

This study provides critical insights into the persistence and transmission of *P. mirabilis* in dairy products, highlighting the urgent need for improved hygiene practices in dairy production, biofilm‐disrupting interventions and enhanced surveillance programs to mitigate risks to animal and public health.

## 2 Methods

### 2.1 Ethical Approval and Consent to Participate

The study was ethically approved by the Ethical Committee of the Islamic Azad University, Shahrekord Branch (Approval Code: IR.IAU.SHK.REC.1402.154). All participants provided written informed consent before sample collection. Ethical considerations included ensuring anonymity, data confidentiality and adherence to the principles outlined in the Declaration of Helsinki.

### 2.2 Study Design and Sampling

This cross‐sectional descriptive study was conducted between September and December 2023 in Shahrekord, located in Chaharmahal and Bakhtiari Province, Iran, to investigate antibiotic resistance and biofilm formation in *P. mirabilis* isolates from dairy products. A total of 480 samples were randomly collected from 25 retail outlets and supermarkets across different districts of Shahrekord to ensure geographic diversity and minimize sampling bias.

The sample types included raw cow, sheep, and goat milk (*n* = 180), traditional cheese (*n* = 60), cream (*n* = 60), dough (*n* = 60), yogurt (*n* = 60) and curd (*n* = 60). Products were selected based on the following inclusion criteria: fresh, unexpired, commercially distributed (either packaged or bulk) and visibly free from spoilage or contamination.

All samples were transported in cold chain conditions (temperature maintained below 4°C) and delivered to the microbiology laboratory of the Islamic Azad University, Shahrekord Branch, within 4 h of collection. The required sample size was calculated using a 95% confidence level, 5% margin of error and an expected prevalence of 20%, based on similar previous studies (Tribst et al. 2020).

### 2.3 Isolation and Identification of *P. mirabilis*

Solid samples (e.g., cheese, cream, yogurt) were homogenized in 90 mL of Trypticase Soy Broth (TSB, Merck, Germany) using a stomacher under sterile conditions. Liquid samples (e.g., milk, dough) were directly inoculated into TSB. After enrichment at 37°C for 24 h, 300 µL of the broth was streaked onto Xylose Lysine Deoxycholate (XLD) agar (Merck, Germany) and incubated for 24 h at 37°C. Colonies with black centres were presumed to be *P. mirabilis* and further identified using swarming motility, IMViC tests, urease activity, and maltose fermentation. Quality control included *P. mirabilis* (ATCC12453) and *Klebsiella pneumoniae* (ATCC700603) as reference strains (Kim et al. 2005).

### 2.4 Antimicrobial Susceptibility Testing

Antimicrobial susceptibility was assessed using the disk diffusion method on Mueller–Hinton agar (Condalab, Spain), following Clinical and Laboratory Standards Institute (CLSI 2022) guidelines. The antibiotics tested included cotrimoxazole, amikacin, nitrofurantoin, cefotaxime, ceftazidime, ceftriaxone, ciprofloxacin and tetracycline, among others. Zones of inhibition were measured and interpreted per CLSI standards.

### 2.5 Detection of ESBL‐Producing Strains

Extended‐spectrum β‐lactamase (ESBL)‐producing strains were identified using the double disk‐diffusion synergy test (DDST). *P. mirabilis* isolates were inoculated onto Mueller–Hinton agar. Cefotaxime (30 µg) and ceftazidime (30 µg), alone and combined with clavulanic acid (30/10 µg), were placed 20 mm apart. A ≥ 5 mm increase in the zone diameter with clavulanic acid confirmed ESBL production.

### 2.6 Biofilm Formation Assessment

Biofilm production was quantified using the microtiter plate assay (Djordjevic et al. 2002). After incubation, wells were washed, stained with crystal violet, and biofilm biomass was measured spectrophotometrically at 570 nm. *Klebsiella pneumoniae* ATCC 1705 served as the positive control.

### 2.7 Molecular Detection of Resistance Genes

Resistance genes, including *qnrA*, *qnrB*, *tetA*, *tetB*, *Sul1* and β‐lactamase genes (*blaCTX‐M*, *blaSHV*, *blaTEM*), were detected using PCR (Ali et al. 2014; Michelim et al. 2008). Primers and amplification conditions are summarized in Table 1. PCR products were visualized on 1.5% agarose gels containing ethidium bromide, and band sizes were compared against a 100 bp molecular marker (Fermentas, Germany).

**TABLE 1:** PCR conditions and primers for resistance genes detection in *Proteus mirabilis*.

Gene	Primers (5′–3′)	PCR cycles	Amplicon (bp)
*ureR*	F: GGTGAGATTTGTATTAATGG R: ATAATCTGGAAGATGACGAG	95°C 5 min, 32 × (94°C 60 s, 60°C 60 s, 72°C 2 min), 72°C 5 min	225
*qnrA*	F: CAGCAAGAGGATTTCTCACG R: AATCCGGCAGCACTATTACTC	94°C 5 min, 32 × (94°C 60 s, 58°C 60 s, 72°C 2 min), 72°C 5 min	360
*qnrB*	F: GGCTGTCAGTTCTATGATCG R: GAGCAACGATGCCTGGTAG	95°C 5 min, 30 × (95°C 30 s, 59°C 30 s, 72°C 60 s), 72°C 6 min	488
*qnrS*	F: GCAAGTTCATTGAACAGGGT R: TCTAAACCGTCGAGTTCGGCG	95°C 5 min, 30 × (95°C 30 s, 59°C 30 s, 72°C 60 s), 72°C 6 min	428
*tetA/B*	F: GTAATTCTGAGCACTGTCGC R: CTGCCTGGACAACATTGCTT	95°C 5 min, 30 × (95°C 30 s, 60°C 30 s, 72°C 60 s), 72°C 6 min	927/659
*ant(2/3)Ia*	F: CGCCGTGGGTCGATGTTTGATG R: TTTTCCGCCCCGAGTGAGGTG	95°C 5 min, 30 × (95°C 30 s, 57°C 30 s, 72°C 60 s), 72°C 6 min	572/245
*blaSHV/CTX‐M/TEM*	F: ATGCGTTATATTCGCCTGTG R: TGCTTTGTTATTCGGGCCAA	95°C 5 min, 30 × (95°C 30 s, 58°C 30 s, 72°C 60 s), 72°C 6 min	747/593/445
*Sul1*	F: CGGCGTGGGCTACCTGAACG R: GCCGATCGCGTGAAGTTCCG	95°C 5 min, 30 × (95°C 30 s, 57°C 30 s, 72°C 60 s), 72°C 6 min	443

*Note*: PCR mix (50 µL): Buffer (5 µL), MgCl_2_ (2 mM), dNTPs (200 µM), primers (0.4–1 µM), Taq polymerase (1–2 U), DNA template (2.5–3 µL). Cycle conditions vary slightly per target gene.

### 2.8 Statistical Analysis

Data were analysed using SPSS version 25.0 (IBM, USA). Fisher's exact test was used for categorical data, and logistic regression was applied to evaluate predictors of resistance. A *p* value of < 0.05 was considered statistically significant.

## 3 Results

### 3.1 Identification and Prevalence of *P. mirabilis*

Biochemical and molecular analyses confirmed the presence of *P. mirabilis* in 59 out of 480 dairy product samples, resulting in an overall prevalence rate of 12.29%. Among the tested dairy products, cow's milk exhibited the highest contamination rate (21.66%), followed by goat milk (20.00%) and sheep milk (18.33%). Conversely, curd displayed the lowest prevalence (5.08%). Statistical analysis demonstrated a significant association between the type of dairy product and *P. mirabilis* contamination (*χ* ^2^ = 115.86, *p* value < 0.05).

**FIGURE 1:**
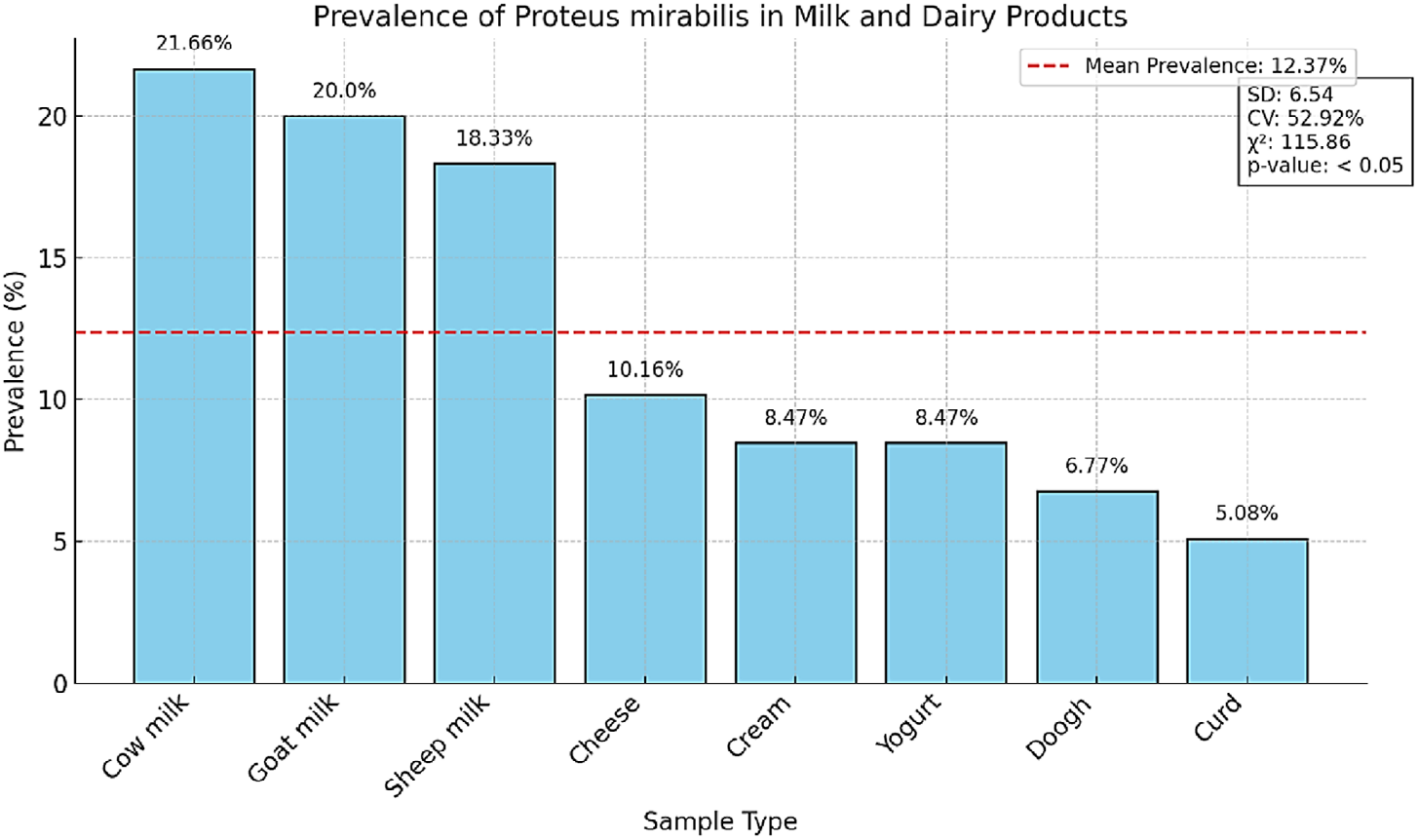
Prevalence of *Proteus mirabilis* in milk and dairy products.

A detailed summary of prevalence rates is presented in Table 1, while Figure 1 graphically illustrates the data.

The statistical analysis demonstrated that the mean prevalence of *P. mirabilis* contamination in dairy products was 12.37%, with a standard deviation (SD) of 6.54%, reflecting variability in contamination levels across different samples. The coefficient of variation (CV) was calculated at 52.92%, indicating substantial variability in contamination risks among the tested dairy products. This high CV suggests that certain products, such as raw cow's milk, are significantly more prone to contamination compared to others like curd or cheese.

The Chi‐square test revealed a statistically significant association between the type of dairy product and the prevalence of *P. mirabilis* contamination (*χ* ^2^ = 115.86, *p* value < 0.05). This finding highlights the importance of implementing product‐specific interventions, particularly for raw milk, which exhibited the highest contamination rate at 21.66%. The significant association underscores the need for targeted hygiene practices, stringent quality control measures during milk collection and processing, and enhanced monitoring protocols to effectively mitigate contamination risks.

#### 3.1.1 Key Observations

1. *Raw cow's milk*: With a contamination rate of 21.66%, raw cow's milk emerged as the most susceptible product, necessitating immediate attention to improve hygiene and processing standards.
2. *Variability in contamination*: The high CV (52.92%) underscores the significant differences in contamination risks across dairy products, emphasizing the need for tailored interventions based on product type.
3. *Implications for dairy production*: The findings highlight the critical role of targeted quality control measures, particularly for high‐risk products like raw milk, to ensure food safety and reduce public health risks associated with *P. mirabilis* contamination.

### 3.2 Molecular Identification of the ureR Gene in *P. mirabilis*

To confirm the presence of the ureR gene in *P. mirabilis* isolates, polymerase chain reaction (PCR) was performed using specific primers designed to amplify a 225 bp fragment of the gene. A total of 59 isolates were subjected to this analysis, and the results demonstrated high consistency in detecting the ureR gene among the positive isolates.

**FIGURE 2:**
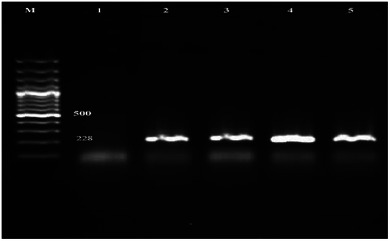
PCR amplification of the ureR gene in *Proteus mirabilis* isolates. Lane M: DNA size marker (100 bp ladder). Lane 1: Negative control. Lane 2: Positive control. Lanes 3–5: Positive isolates showing 225 bp amplicons.

Figure 2 **presents** the electrophoretic analysis of the PCR products on a 1.5% agarose gel. The DNA ladder (100 bp) was used as a molecular size marker. A negative control (Lane 1) and a positive control (Lane 2) were included to validate the specificity of the amplification. Lanes 3 to 5 represent PCR‐positive samples with distinct amplicons at the expected size of 225 bp, confirming the presence of the ureR gene.

#### 3.2.1 Analysis and Significance

The amplification of the ureR gene in these isolates highlights its prevalence among the *P. mirabilis* strains isolated from dairy samples. The ureR gene, associated with urease activity, plays a critical role in pathogenicity, particularly in urinary tract infections caused by *P. mirabilis*. The identification of this gene underlines the need for further research into its potential as a target for diagnostic or therapeutic interventions.

These results reinforce the reliability of molecular techniques in the accurate identification of pathogenic strains and provide a basis for advanced genomic studies to explore the role of urease in virulence and antimicrobial resistance.

### 3.3 Antibiotic Resistance Patterns

**FIGURE 3:**
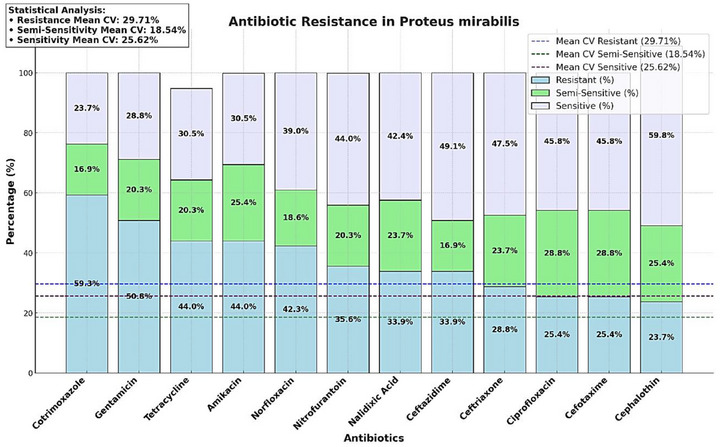
Antibiotic resistance in *Proteus mirabilis*. Statistical analysis: Resistance mean CV: 29.71%. Semi‐sensitivity mean CV: 18.54%. Sensitivity mean CV: 25.62%.

Antibiotic susceptibility testing revealed diverse resistance patterns among the *P. mirabilis* isolates. Resistance rates and variability are summarized in Figure 3.

#### 3.3.1 Analysis

Cotrimoxazole exhibited the highest resistance rate (59.32%), followed by gentamicin (50.84%) and tetracycline (44.00%). Cephalothin, with the lowest resistance rate (23.72%), may remain effective against some strains. The high CV for resistance (29.71%) highlights substantial variability, reflecting the complexity of antimicrobial resistance in *P. mirabilis*. These findings underscore the importance of further genomic studies to elucidate resistance mechanisms and guide antibiotic stewardship programs.

### 3.4 Antibiotic Resistance by Sample Type

**FIGURE 4:**
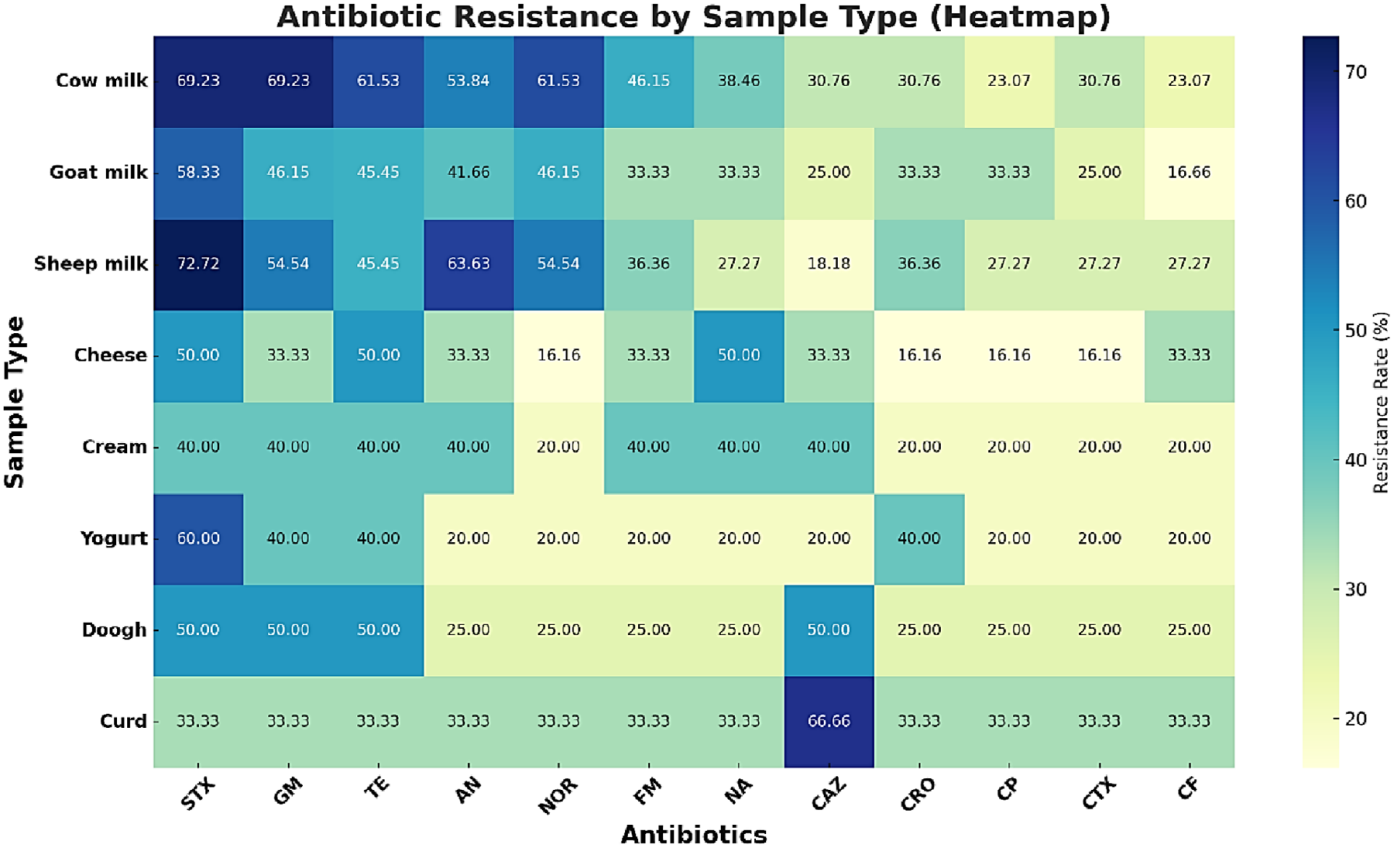
Antibiotic resistance by sample type.

Antibiotic resistance patterns varied significantly among dairy product types, as detailed in Figure 4. Statistical analysis confirmed a significant association between sample type and resistance rates (*p* value < 0.05).

The heatmap displays antibiotic resistance rates (%) among *P. mirabilis* isolates derived from various dairy products, including cow milk, goat milk, sheep milk, cheese, cream, yogurt, doogh (a traditional Iranian fermented yogurt‐based drink) and curd. Each row represents a sample type, and each column corresponds to a specific antibiotic (e.g., STX: cotrimoxazole, GM: gentamicin, TE: tetracycline, etc.). The colour intensity reflects the percentage of antibiotic resistance, with darker shades indicating higher resistance rates.

#### 3.4.1 Key Observations

1. Cow milk exhibited the highest resistance rates for cotrimoxazole (STX, 69.23%) and gentamicin (GM, 69.23%), emphasizing its role as a significant reservoir for resistant strains.
2. Sheep milk demonstrated a similar pattern with elevated resistance to cotrimoxazole (STX, 72.72%) and gentamicin (GM, 54.54%), but moderate levels for other antibiotics.
3. Cheese and curd showed relatively lower resistance levels for most antibiotics, potentially attributable to specific processing methods that reduce bacterial contamination or eliminate resistant strains.
4. Variability in resistance rates highlights the need for targeted interventions based on sample type. For instance, stricter control measures should be implemented for raw milk sources, such as cow and sheep milk, compared to processed dairy products like cheese and curd.

#### 3.4.2 Statistical Significance

- Statistical analysis (*p* value < 0.05) confirmed a significant association between sample type and antibiotic resistance patterns.
- These findings underline the importance of tailoring antibiotic stewardship and food safety strategies for specific dairy product categories.

This heatmap effectively summarizes complex data, offering a clear visual representation of antibiotic resistance variability, making it easier for readers to identify critical trends across multiple sample types and antibiotics.

### 3.5 Biofilm Formation

**FIGURE 5:**
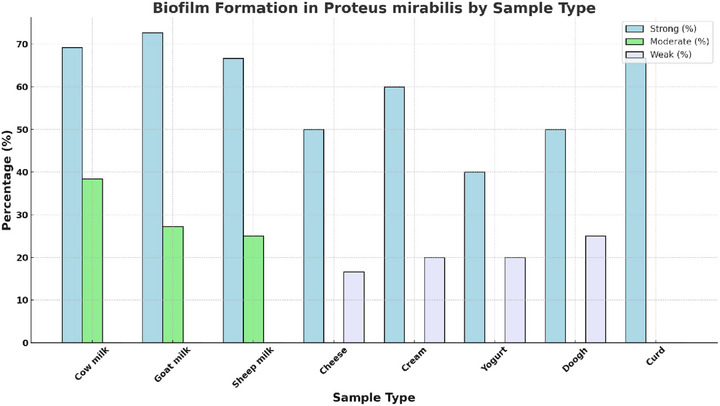
Correlation between biofilm formation and antibiotic resistance.

Among the 59 *P. mirabilis* isolates, biofilm formation was observed in **88.12%** of the samples. Of these, 71.15% exhibited strong biofilm production, 21.15% moderate and 7.69% weak. Statistical analysis indicated a significant association between biofilm formation ability and the type of dairy product (*p* value < 0.05) (Figure 5).

This clustered bar chart illustrates the distribution of biofilm formation (strong, moderate and weak) among *P. mirabilis* isolates across various dairy products. The colours have been selected to provide a calming and professional visual appearance.

#### 3.5.1 Key Observations

1. Strong biofilm formation was most prevalent in raw dairy products, such as cow milk (69.23%) and goat milk (72.72%), indicating their potential as major reservoirs for biofilm‐forming strains.
2. Processed products, such as cheese and yogurt, exhibited lower levels of strong biofilm formation and higher levels of weak biofilm formation, potentially due to processing steps that mitigate microbial persistence.
3. The mean strong biofilm formation across all samples was 59.73%, with a CV of 14.7%, reflecting moderate variability.

#### 3.5.2 Statistical Analysis

- A statistically significant association (*p* value < 0.05) was observed between the type of dairy product and biofilm formation capabilities.
- These results emphasize the dual challenge of biofilm formation and antimicrobial resistance in raw dairy products, necessitating targeted contamination control strategies.

These findings underscore the dual challenge of antibiotic resistance and biofilm formation in *P. mirabilis*, particularly in raw dairy products like cow and goat milk. Integrated control measures targeting contamination sources and microbial persistence are essential for mitigating these risks.

### 3.6 Correlation Between Biofilm Formation and Antibiotic Resistance

**FIGURE 6:**
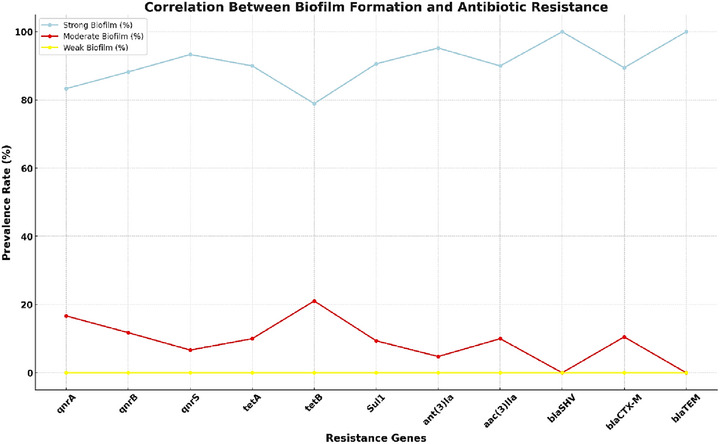
Correlation between biofilm formation and antibiotic resistance.

A strong correlation was observed between biofilm formation strength and antibiotic resistance. Isolates with strong biofilm‐forming capabilities exhibited significantly higher prevalence rates of antibiotic resistance genes. Statistical analysis confirmed this relationship (*χ* ^2^ = 49.56, *p* value < 0.05) (Figure 6).

This line chart illustrates the prevalence rates of antibiotic resistance genes in *P. mirabilis* isolates based on biofilm formation strength (strong, moderate, weak). Each line represents a biofilm strength category, allowing for a clear comparison of resistance gene prevalence across biofilm levels.

#### 3.6.1 Key Observations

1. Strong biofilm producers consistently showed high prevalence rates for resistance genes, particularly blaSHV and blaTEM (100%) and ant(3)Ia (95.23%).
2. Moderate biofilm producers displayed significantly lower prevalence rates, ranging from 4.76% to 21.05%, depending on the gene.
3. Weak biofilm producers exhibited no detectable resistance genes, emphasizing the role of biofilm strength in antimicrobial resistance.

#### 3.6.2 Advantages of This Visualization

- The line chart provides a clear and simple comparison of resistance prevalence across genes and biofilm categories.
- The use of gridlines and distinct markers enhances readability and makes trends easier to follow.
- This format highlights the strong correlation between biofilm formation strength and resistance.

### 3.7 ESBL Production and Beta‐Lactamase Genes

**FIGURE 7:**
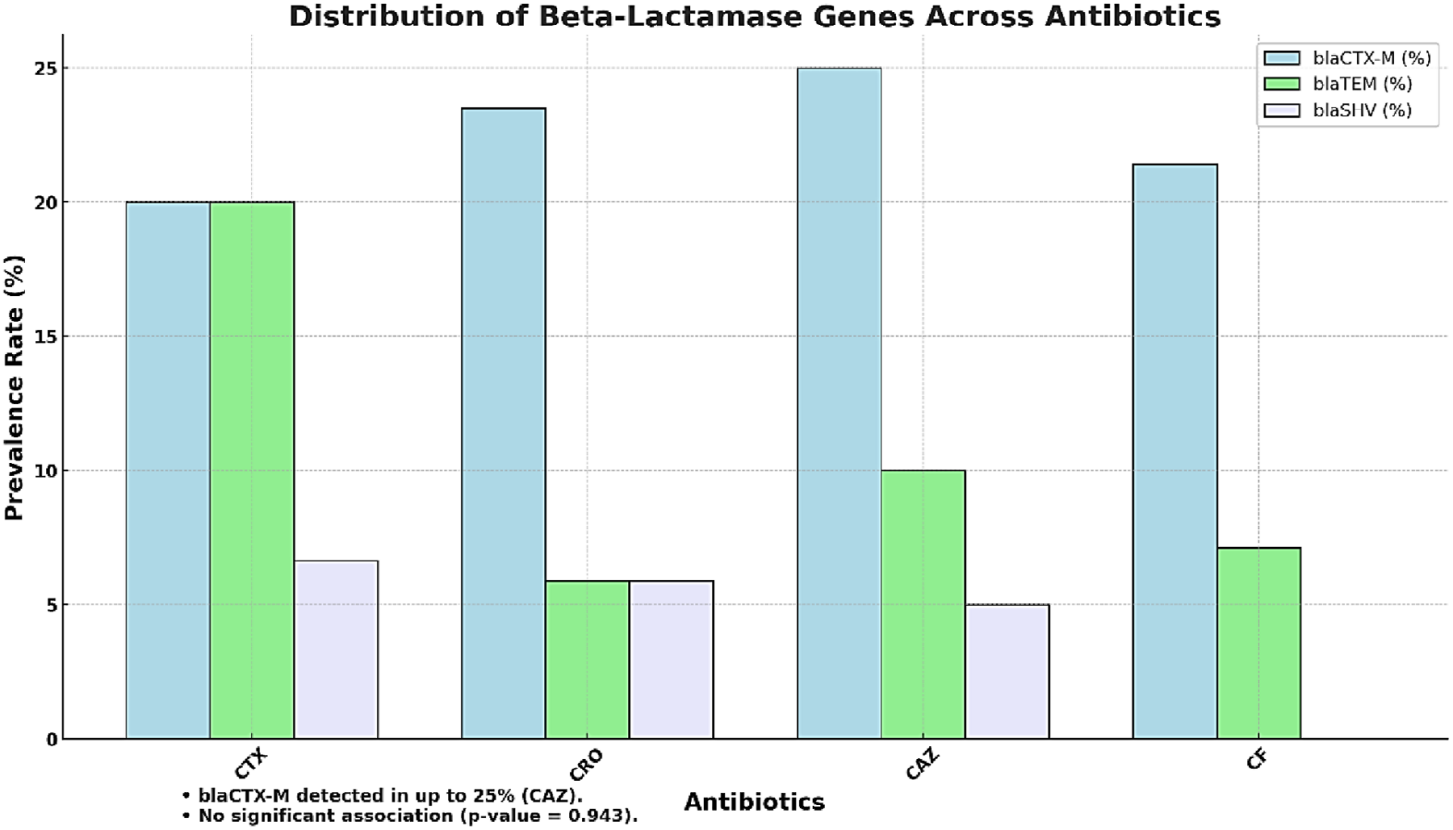
Distribution of beta‐lactamase genes across antibiotics.

Phenotypic testing identified 15 out of 59 *P. mirabilis* isolates (25.42%) as extended‐spectrum beta‐lactamase (ESBL) producers. The distribution of beta‐lactamase genes across beta‐lactam antibiotics is summarized in Figure 7. Statistical analysis revealed no significant association between specific beta‐lactamase genes and the antibiotics tested (*p* value = 0.943).

This clustered bar chart shows the prevalence of beta‐lactamase genes (blaCTX‐M, blaTEM, blaSHV) among *P. mirabilis* isolates tested with beta‐lactam antibiotics (CTX, CRO, CAZ, CF). Statistical analysis summary is displayed below the chart.

#### 3.7.1 Key Observations

- blaCTX‐M was the most prevalent gene, detected in up to 25% of isolates tested with CAZ.
- blaTEM and blaSHV were less frequently observed, with blaSHV absent in isolates tested with CF.
- No significant association was found between beta‐lactamase genes and specific antibiotics (*p* value = 0.943).

### 3.8 Comprehensive Overview of Antibiotic Resistance Genes

**FIGURE 8:**
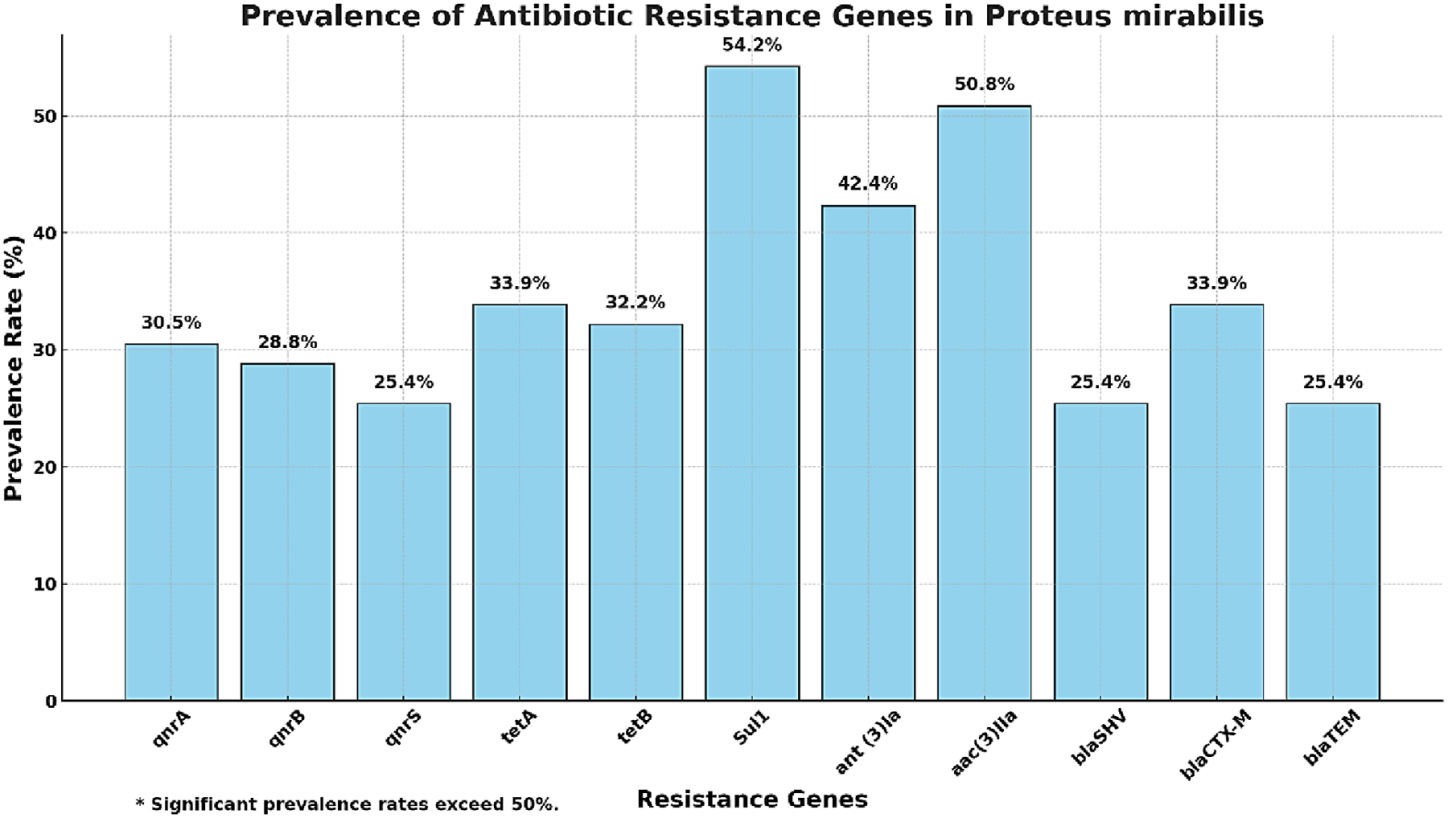
Prevalence of antibiotic resistance genes in *Proteus mirabilis*.

The prevalence of resistance genes across all *P. mirabilis* isolates is summarized in Figure 8. Sul1 (54.23%) and aac(3)IIa (50.84%) were the most frequently detected, while qnrS (25.42%) and qnrB (28.81%) were the least common.

This bar chart displays the prevalence rates of various antibiotic resistance genes in *P. mirabilis* isolates. Each bar is labelled with its respective percentage for clarity.

## 4 Discussion

### 4.1 Prevalence and Comparative Epidemiology

In the present study, *P. mirabilis* was isolated from 12.29% of raw milk and dairy products, with cow's milk displaying the highest contamination rate (21.66%). These findings are in close agreement with those of El‐Maghraby et al. (2024), who reported a 12.67% prevalence in dairy samples from Egypt, particularly in raw milk and Kariesh cheese. This similarity may reflect comparable handling conditions and public exposure to open‐air dairy marketing (El‐Maghraby et al. 2024). In contrast, a significantly higher prevalence (25.65%) was reported in retail meat and aquatic products in China, indicating potential differences in food matrices and sanitation standards (Ma et al. 2022).

### 4.2 Antimicrobial Resistance and Gene Profiles

Our findings revealed high resistance to cotrimoxazole and penicillin‐class antibiotics, paralleling previous studies in Egypt and China. El‐Maghraby et al. (2024) reported 100% resistance to erythromycin and 94.7% to tetracycline, while Ma et al. (2022) identified multidrug resistance (MDR) in 91.01% of *P. mirabilis* strains isolated from food markets. Notably, shared resistance genes including *blaCTX‐M*, *aac(6′)‐Ib‐cr* and *sul1* were prevalent across studies, highlighting a consistent global dissemination of these resistance determinants. These findings underscore the dairy chain as a reservoir of antimicrobial resistance genes (ARGs), which can be transmitted to human pathogens.

### 4.3 Biofilm Formation and Virulence Determinants

The current study showed a strong correlation between biofilm formation and resistance phenotypes. This observation aligns with molecular investigations by El‐Maghraby et al. (2024), who detected high frequencies of virulence genes such as *luxA*, *flaA*, *mrpA* and *hpmA*, which are implicated in bacterial adhesion, motility and biofilm maturation. These virulence traits increase the pathogen's survival in dairy environments and contribute to chronic contamination risks.

### 4.4 Microbial Ecology and Horizontal Gene Transfer

The global expansion of ARGs is largely driven by horizontal gene transfer mechanisms. As Voidarou and Tzora (2025) emphasize, microbial diversity enhances the genetic plasticity of bacterial populations, enabling rapid acquisition and dissemination of resistance genes. Our findings support this view, particularly given the identification of mobile genetic elements associated with *blaNDM‐1*, *tmexCD3‐toprJ1* and *cfr* in food‐associated isolates (Ma et al. 2022). Such ecological interactions highlight the role of dairy products in mediating ARG mobility across microbial communities.

### 4.5 One Health Perspective and Public Health Implications

The high prevalence of MDR *P. mirabilis* in dairy products poses a direct risk to public health through consumption or cross‐contamination (Sanches et al. 2019; Li et al. 2022; Yeh et al. 2023; Yu et al. 2020). As Ma et al. (2022) and Voidarou and Tzora (2025) articulate, the intersection of food production, human health and environmental microbiota necessitates a One Health strategy. The identification of ESBL and aminoglycoside resistance genes in foodborne *P. mirabilis* underscores the urgent need for integrated surveillance and antimicrobial stewardship.

### 4.6 Regional Discrepancies and Methodological Considerations

Discrepancies in prevalence and resistance profiles among studies can be attributed to differences in sampling methods, local antimicrobial usage and sanitation practices. While our data are consistent with the Egyptian dairy context (El‐Maghraby et al. 2024), they diverge from some aspects of Chinese data, particularly in the diversity and mobility of resistance genes (Ma et al. 2022). These differences highlight the necessity for harmonized methodologies in food safety research.

### 4.7 Implications for Risk Management

The findings support immediate action in three critical areas: (1) the application of biofilm‐targeted interventions such as quorum‐sensing inhibitors and enzymatic disruptors; (2) reinforcement of hygiene protocols during milk production and handling; and (3) development of national policies for antibiotic usage in food animals. These interventions, alongside molecular surveillance, will help contain the spread of ARGs.

### 4.8 Future Directions

This study contributes to the growing body of evidence on the role of *P. mirabilis* in foodborne antimicrobial resistance. Future research should investigate the genetic linkage between virulence and resistance traits and explore the efficacy of novel disinfection technologies in dairy processing environments.

## 5 Conclusion

This study underscores the significant risk posed by *P. mirabilis* in dairy products, particularly raw milk, which exhibited the highest contamination rates. The strong correlation between biofilm formation and antibiotic resistance highlights the bacterium's resilience and its potential to act as a reservoir for resistance genes, raising serious public health concerns.

Addressing these risks requires stricter hygiene protocols in dairy production, targeted interventions to disrupt biofilms and surveillance programs to monitor antimicrobial resistance. By identifying key resistance and biofilm‐associated genes, this research lays a foundation for developing strategies to enhance food safety and mitigate public health threats.

## Author Contributions

**Afrooz Shafiei Lordejani**: software, formal analysis, data curation, validation, writing – original draft. **Elaheh Tajbakhsh**: conceptualization, investigation, writing – original draft, methodology, writing – review and editing, software, formal analysis, project administration, visualization. **Faham Khamesipour**: methodology, validation, project administration, visualization. **Hassan Momtaz**: methodology, formal analysis, software. **Manocher Momeni Shahraki**: methodology, visualization, writing – review and editing.

## Ethics Statement

All procedures performed in this study complied with ethical standards.

## Conflicts of Interest

The authors declare no conflicts of interest.

## Peer Review

The peer review history for this article is available at https://www.webofscience.com/api/gateway/wos/peer‐review/10.1002/vms3.70600.

## Data Availability

Data supporting the findings of this study are available upon reasonable request by contacting the corresponding author via email.
